# Research on Depression Recognition Model and Its Temporal Characteristics Based on Multiscale Entropy of EEG Signals

**DOI:** 10.3390/e27020142

**Published:** 2025-01-31

**Authors:** Xin Xu, Jiangnan Xu, Ruoyu Du, Tingting Xu

**Affiliations:** School of Communication and Information Engineering, Nanjing University of Posts and Telecommunications, Nanjing 210003, China; xuxin@njupt.edu.cn (X.X.); 1022010409@njupt.edu.cn (J.X.); xutt@njupt.edu.cn (T.X.)

**Keywords:** depression, electroencephalogram, temporal scale, multiscale entropy, machine learning

## Abstract

The diagnosis of depression is a critical topic in the medical field. For years, the electroencephalogram (EEG) has been considered an objective and cost-effective detection tool. However, most studies on depression recognition models tend to extract information solely from the original temporal scale of EEG signals, ignoring the usage of coarse scales. This study aims to explore the feasibility of multiscale analysis for a depression recognition model and to research its temporal characteristics. Based on two types of multiscale entropy, this paper constructs a machine learning model using classifiers including LDA, LR, RBF-SVM, and KNN. The relation between the temporal scale and model performance was examined through mathematical analysis. The experimental results showed that the highest classification accuracy achieved was 96.42% with KNN at scale 3. Among various classifiers, scales 3 and 9 outperformed other scales. The model performance is correlated with the scale variation. Within a finite range, an optimal scale likely exists. The algorithm complexity is linearly related to the temporal scale. By accepting predictable computational costs, a stable improvement in model performance can be achieved. This multiscale analysis is practical in building and optimizing a depression recognition model. Further investigation of the relation between the temporal scale and model capabilities could advance the application of computer-assisted diagnosis.

## 1. Introduction

Nowadays, depression is one of the most common mental illnesses. According to a report from the World Health Organization, more than 264 million people worldwide suffer from depression, and approximately 35.8% of suicides are attributed to depression [1]. Currently, there is no effective treatment for depression. It is important to screen the potential population and intervene at the early stages of the illness. The international standards for the diagnosis of depression are the Diagnostic and Statistical Manual of Mental Disorders IV (DSM-IV) and a clinical test called the Mini-Mental State Examination (MMSE). However, due to variations in doctors’ experience levels and patients’ individual conditions, depression diagnoses are often subjective, resulting in low accuracy and poor consistency, which may lead to misdiagnosis. In recent years, many studies using electroencephalogram (EEG) signals to detect depression have emerged. Compared to other brain imaging methods, such as computed tomography (CT) or functional magnetic resonance imaging (fMRI), EEG equipment offers advantages in terms of lower costs and shorter detection times, making it suitable for the identification of mental illnesses in routine clinical settings [2].

The core of the depression recognition process based on EEG is to extract signal parameters (biomarkers) that can effectively characterize the pathology. By quantifying these biomarkers, machine learning models can be trained to identify depression in potential patients. In order to search for effective biomarkers, researchers have performed many studies on the time domain, frequency domain, spatial domain, or nonlinear domain. However, most of these studies were conducted at a single temporal scale. For physiological signals, analysis at a single scale may lead to the misjudgment of system complexity. Therefore, Costa et al. [3] proposed a multiscale method and introduced the concept of multiscale entropy (MSE). The basic idea is to coarse-grain a time series in order to obtain the information present in multiple temporal scales. Several studies have been conducted on the application of MSE. Angsuwatanakul et al. [4] used MSE to analyze the complexity of brain activity. Fifteen healthy subjects participated in a visual memory task. The experimental results showed that the level of brain complexity in the prefrontal and frontal lobes of the subjects who intentionally remembered a visual scene was significantly higher than that in the subjects who intentionally forgot the scene. Zou et al. [5] applied MSE to fatigue detection under driving situations. The classification accuracy using multiscale fuzzy entropy was up to 88.74%, which was 23.88% higher than that of the single-scale fuzzy entropy method. Jaworska et al. [6] investigated the relationship between local (fine scale) and global (coarse scale) neural processing measured before antidepressant treatment and the subsequent treatment response. A reduction in MSE at fine scales and an increase in MSE at coarse scales were associated with the degree of antidepressant response. Yun et al. [7] studied the specific temporal scale range that characterized major depressive disorder (MDD) by measuring the refined composite multiscale permutation entropy (RCMPE) [8] during a resting state. Compared to healthy controls, the EEG variability in MDD patients only increased at coarse scales. Variability at the fine scales was inversely associated with the severity of depressive symptoms. These characteristics have not been found using traditional analysis methods, such as the neural oscillation power.

According to the current research, the multiscale method has been applied in some EEG studies and proven effective. However, whether it could be used for depression recognition remains to be determined. This work aims to explore the feasibility of multiscale analysis for depression recognition models and to research its temporal characteristics.

The main contributions of this study are summarized as follows:

- The feasibility of applying EEG-based multiscale analysis to depression recognition is demonstrated;
- The machine learning models are trained and verified on various temporal scales and classifiers to select the optimal configurations;
- The relation between model performance/complexity and temporal scale variation is investigated.

## 2. Materials and Methods

### 2.1. Dataset Description

In this study, EEG data provided by the Hospital University Sains Malaysia (HUSM) were used for signal analysis. According to the experimental design, approved by the human ethics committee of HUSM, 34 outpatients with MDD were recruited, including 17 males and 17 females, with an average age of 40.3 ± 12.9 years. Meanwhile, 30 age-matched healthy controls were recruited, including 21 males and 9 females, with an average age of 38.3 ± 15.6 years. All subjects fully understood the experimental content and signed a consent form. The MDD patients enrolled met the internationally recognized diagnostic criteria for depression, known as the DSM-IV [9].

The EEG acquisition equipment followed the international 10-20 system. Data were recorded with the Linked Ear (LE) reference and digitized at 256 Hz. The pre-filter range was 0.1–70 Hz, with a 50 Hz notch filter to suppress power line noise. Resting-state data with eyes closed were used for our study. In this paradigm, the subjects were instructed to sit in a semi-rectifying position with minimal head movement, recording for 5 min when the eyes were closed.

### 2.2. Pre-Processing

The EEGLAB toolbox in MATLAB was used to pre-process the raw data. Firstly, channel location was performed according to the international 10-20 head electrode model. Then, band pass filtering of 0.1–45 Hz was carried out. Next, the Clean_Rawdata tool provided with EEGLAB was utilized for bad segment removal. Then, based on the procedure of independent component analysis (ICA), noise and artifact components were screened out by experts [10].

### 2.3. Multiscale Analysis

**Figure 1:**
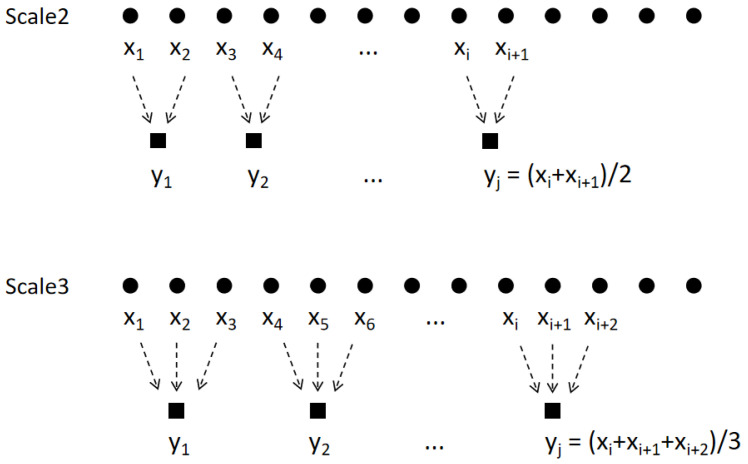
The basic idea of multiscale analysis: coarse-graining. *x* is the original sequence. The successive values of *x* are averaged to obtain the reconstructed sequence *y*. The concept of the temporal scale corresponds to the number of successive points; it can also be understood as the window length.

Multiscale analysis is derived from the method proposed by Costa et al. [3]. The basic idea is to coarse-grain a sequence, as shown in Figure 1.

To conduct an analysis with a temporal scale of 2, each successive 2 sample points of the original time series are replaced by their mean. For an analysis with a temporal scale of 3, each of the 3 consecutive sample points of the original time series are replaced by their mean. Similarly, when analyzing with a temporal scale of τ, every successive τ sample points of the original time series are replaced by the mean value. This process is called coarse-graining. The new sequence after coarse-graining has the form

(1) $$y_{j}^{\left(\tau \right)}=\frac{1}{\tau }\sum_{i=(j-1)\tau +1}^{j\tau }x_{i},1\leq j\leq \frac{N}{\tau },$$

where *x* is the original sequence and *y* is the sequence after coarse-graining, also denoted as the reconstruction sequence. τ represents the scale factor.

The entropy calculated for the reconstructed sequence (e.g., sample entropy [11], permutation entropy [12]) is called multiscale entropy at scale τ, which is the most primitive multiscale algorithm. Note that the length of a reconstructed sequence is 1/τ that of the original sequence. This means that when the temporal scale τ is set at a large value, the reconstructed sequence will not be long enough to provide a reliable entropy estimation. In addition, an extremely short length may lead to undefinable entropy (considering that there is no matching template vector to be found in the sample entropy algorithm [11]). Thus, several improved algorithms have been proposed. The following describes two of these approaches, which are used to calculate the sample entropy and permutation entropy, respectively, under the multiscale perspective.

### 2.4. Refined Composite Multiscale Sample Entropy (RCMSE)

Wu et al. [13] proposed the RCMSE algorithm. Based on the concept of the original sample entropy, the number of matching vectors under different temporal scales is averaged; in this way, we can simultaneously exploit the information of the series at all scales and prevent the occurrence of zero in the numerator or denominator when calculating the logarithm. The flow of the RCMSE algorithm is described as follows.

1. Specify a temporal scale τ and calculate the reconstruction sequence on each scale from 1 to τ according to (1), denoted as {$y^{\left(k\right)}$}, where k∈[1,τ].
2. According to the definition of traditional sample entropy [11], the numbers of matching vectors of $y^{\left(k\right)}$ are calculated for each temporal scale *k*, denoted as $n_{m}^{k}$ and $n_{m+1}^{k}$, where *m* is the embedding dimension.
3. Let ${\bar{n}}_{m}^{\tau }$ and ${\bar{n}}_{m+1}^{\tau }$ represent the mean of the matching vectors over all scales, i.e.,
  $${\bar{n}}_{m}^{\tau }=\frac{1}{\tau }\sum_{k=1}^{\tau }n_{m}^{k}$$
  $${\bar{n}}_{m+1}^{\tau }=\frac{1}{\tau }\sum_{k=1}^{\tau }n_{m+1}^{k}$$
4. Following the concept of sample entropy, the RCMSE is defined as
  (2) $$\begin{array}{cc} RCMSE(x,\tau ,m,r) & =-ln(\frac{{\bar{n}}_{m+1}^{\tau }}{{\bar{n}}_{m}^{\tau }})\\ \; & =-ln(\frac{\sum_{k=1}^{\tau }n_{m+1}^{k}}{\sum_{k=1}^{\tau }n_{m}^{k}}), \end{array}$$
  where *x* is the original sequence, τ is the scale factor, *m* is the embedding dimension, and *r* is the noise threshold. According to (2), the entropy value will be undefined only when $n_{m}^{k}$ and $n_{m+1}^{k}$ both take zero at all scales. Thus, RCMSE overcomes a key flaw in the original multiscale method. In our experiment, *m* was set to 2 and *r* was set to 0.15σ (σ represents the standard deviation of a signal), which is a commonly used parameter combination for sample entropy in physiological signal research [4,14].

### 2.5. Refined Composite Multiscale Permutation Entropy (RCMPE)

Referring to the idea of RCMSE, Humeau-Heurtier et al. [8] proposed RCMPE, which also aims to solve the problem of inaccurate entropy estimation in short sequences. The RCMPE algorithm flow is described as follows.

1. Specify a temporal scale τ and calculate the reconstruction sequence on each scale from 1 to τ according to (1), denoted as {$y^{\left(k\right)}$}, where k∈[1,τ].
2. According to the definition of traditional permutation entropy [12], the frequency sets of $y^{\left(k\right)}$ are calculated for each temporal scale *k*, denoted as {$p_{m}^{k}\left(\pi \right)$}, where *m* is the embedding dimension and π is the possible arrangement pattern. The number of patterns is up to *m*!.
3. Let {${\bar{p}}_{m}^{\tau }\left(\pi \right)$} represent the mean of the frequency sets over all scales, i.e.,
  $${\bar{p}}_{m}^{\tau }\left(\pi \right)=\frac{1}{\tau }\sum_{k=1}^{\tau }p_{m}^{k}\left(\pi \right)$$
4. Following the concept of permutation entropy, the RCMPE is defined as
  (3) $$\begin{array}{cc} RCMPE(x,\tau ,m) & =-\sum_{i=1}^{m!}{\bar{p}}_{m}^{\tau }\left(\pi \right)ln\left({\bar{p}}_{m}^{\tau }\left(\pi \right)\right), \end{array}$$
  where *x* is the original sequence, τ is the scale factor, and *m* is the embedding dimension. It is appropriate to set m∈[3,7] according to established theories [15,16]. In this study, *m* was specified as 4, corresponding to 4!=24 possible permutation patterns, which can reduce the calculation cost and retain the reliability of entropy estimation.

Combining (2) and (3), the essential idea of refined class algorithms is to perform the multiscale averaging of the intermediate parameters, in order to minimize the estimation error caused by the short length of the reconstructed sequence.

### 2.6. Feature Construction

Cuesta et al. [17] proposed the entropy complementarity hypothesis, that is, sample entropy and permutation entropy represent information at different levels of the biomedical signal, which could be integrated when characterizing a system. This study concatenated RCMSE and RCMPE to form a feature vector. Specifically, the pre-processed signal was segmented by rectangular windows with a length of 10 s [18]. Then, feature extraction with the two entropy algorithms was carried out in each epoch. To investigate the effect of τ, the entropy between the experimental group and control group was calculated under different temporal scales. Referring to the previous work [7], the maximal scale was controlled within 10. In other words, the variable τ was set at a range from 1 to 10.

### 2.7. Classification

Multiple classifiers from machine learning were applied in our research, including linear discriminant analysis (LDA), logistic regression (LR), support vector machine (SVM), and K-nearest neighbors (KNN). For the kernel function selection of SVM, we used the radial basis function (RBF) and set the kernel scale to 6.2. For KNN, we applied the Euclidean distance and set the number of neighboring points to 10.

**Figure 2:**
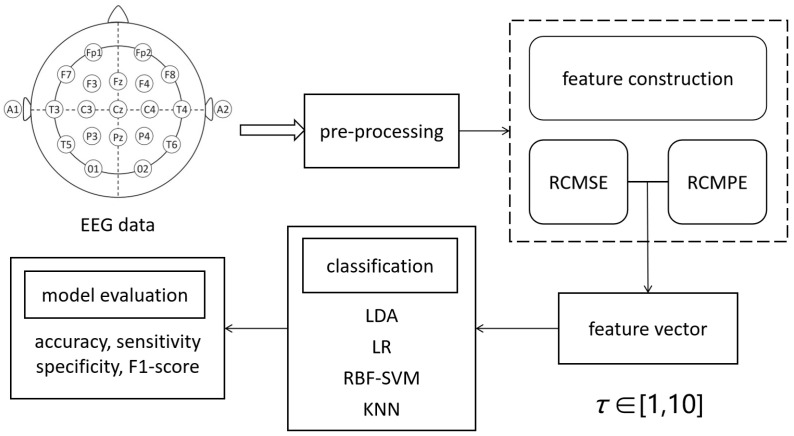
The data flow of depression recognition. The EEG signals are preprocessed first; then, feature extraction is carried out using the incorporation of multiscale entropy to construct a feature vector. The model performance is evaluated under temporal scales from 1 to 10.

Figure 2 is given to illustrate the proposed depression recognition model; it summarizes the research methods mentioned in this section.

### 2.8. Evaluation Metrics

In total, four metrics were used to evaluate the proposed model, including the classification accuracy, sensitivity, specificity, and F1-score, which are defined in (4) to (7).

(4) $$Accuracy=\frac{TP+TN}{TP+FP+FN+TN}$$

(5) $$Sensitivity=\frac{TP}{TP+FN}$$

(6) $$Specificity=\frac{TN}{TN+FP}$$

(7) $$F1\text{-score}=\frac{2TP}{2TP+FP+FN}$$

TP, FP, FN, and TN correspond, respectively, to four prediction cases of the confusion matrix. In this study, statistical model metrics were calculated based on 10-fold cross-validation. The dataset was randomly divided into 10 parts; then, each part was viewed as the testing set, whereas the other 9 parts were viewed as the training set. The average metrics of the 10 experiments were calculated. The validation procedure was completed until all data were treated as the testing set, making the results more reliable.

## 3. Results

The proposed model was constructed for depression classification according to the method described in Section 2. Then, the performance of the obtained model was recorded for τ∈[1,10], as shown in Table 1, Table 2, Table 3 and Table 4, which depict, respectively, the classification accuracy, sensitivity, specificity, and F1-score of the proposed model with different scales and classifiers. The highest classification accuracy was 96.42% when multiscale entropy of scale 9 and the KNN classifier were used. The highest sensitivity was 94.07%, using scale 6 or 8 and the RBF-SVM classifier. The highest specificity was 98.64%, using scale 2 and the RBF-SVM classifier. The highest F1-score was 0.956, with scale 9 and the KNN classifier.

## 4. Discussion

### 4.1. Optimization Analysis

To preliminarily explore the optimal range of the temporal scale, *t*-tests were conducted on the entropy values of HC-MDD signals under different configurations. As shown in Table 5, the group differences in RCMSE were observed at electrodes F3, P3, O1, F7, T3, T5, Fz, Fp2, P4, O2, T4, T6, and Pz. The *p*-value showed a weak correlation with the temporal scale. In contrast, Table 6 indicates that the group differences in RCMPE were influenced by the temporal scale. Specifically, the RCMPE values at scale 1 and scales 7 to 10 exhibited significant differences across all electrodes except T3 and T5. However, at scales 2 to 6, the group differences varied depending on the electrode location. For the same electrode, the influence of scale variation on the group differences was also ambiguous. Given the inconsistency in the statistical results between the two entropy algorithms, the role of the temporal scale cannot be solely determined by means of hypothesis testing. It might be more appropriate to directly investigate the relation between the temporal scale and model evaluation metrics, which could better reflect the joint contribution of the two entropy measurements for depression recognition.

**Figure 3:**
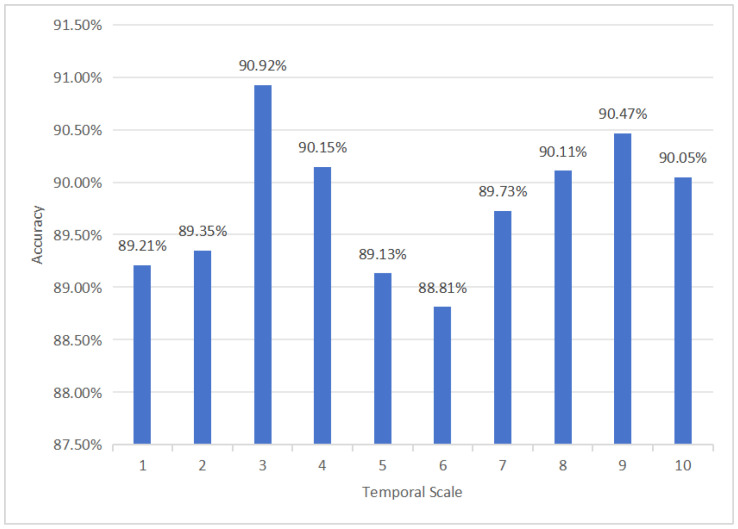
The average accuracy of all classifiers at different temporal scales.

**Figure 4:**
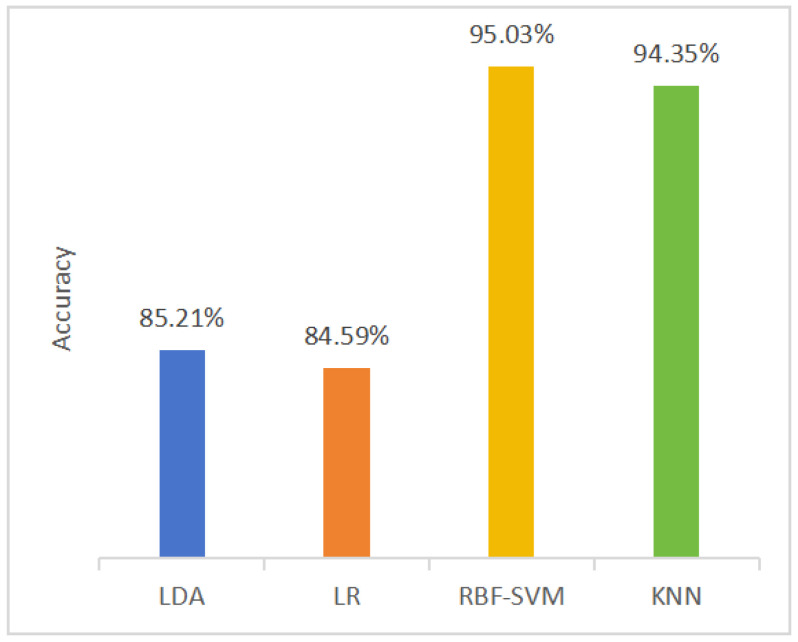
The average accuracy across all temporal scales when using different classifiers.

Taking the accuracy as an example, based on control variates, the model performance was averaged across the classifiers and temporal scales, as shown in Figure 3 and Figure 4.

Figure 3 illustrates that the optimal model performance for all classifiers occurs at scales 3 and 9, with accuracies of 90.92% and 90.47%, respectively. Figure 4 shows that the highest model performance is achieved using the RBF-SVM and KNN classifiers across all scales, with accuracies of 95.03% and 94.35%, respectively. In terms of the performance of the classifier, the results of this study are consistent with previous research [19,20], indicating that SVM could be one of the most effective classifiers for depression recognition. The hyperplane applied in SVM generally exhibits strong class separation capabilities for binary classification tasks involving medium-sized datasets.

Furthermore, to identify the optimal scale, each evaluation metric was considered separately, as summarized in Table 7.

**Figure 5:**
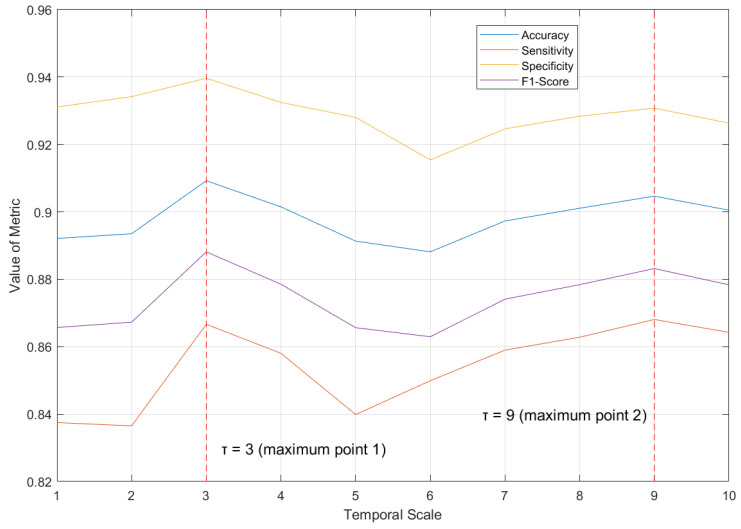
The relation between the average metrics of all classifiers and the temporal scale. As the scale varies, the fluctuation trends of each metric are similar. When τ = 3 or τ = 9, the metric–scale function reaches a local maximum value.

Regardless of whether a single classifier is examined or all classifiers are considered collectively, scales 3 and 9 appear more frequently compared to other scales among the various metrics. A line chart was plotted to depict the fluctuation trend in the model performance across all scales, based on the average metrics of all classifiers, as shown in Figure 5.

**Figure 6:**
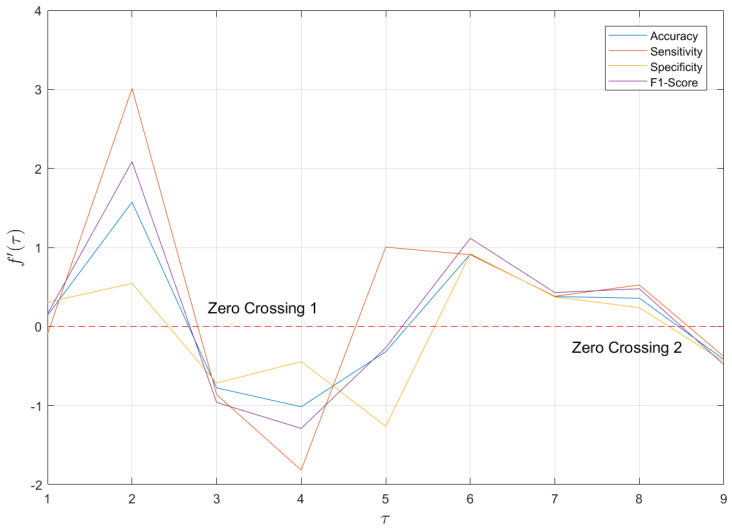
The forward difference function of metric–scale—$f^{\prime }\left(\tau \right)$. Each curve has three zero crossings, two of which are transitions from positive to negative values, corresponding to local maximum points of f(τ).

Combining Table 7 and Figure 5, scales 3 and 9 are not only the points where the model performance reaches its maximum (global extremum) but also appear to be the local extrema with respect to the temporal scale. To verify this hypothesis, the temporal scale is viewed as an independent variable, and the metric is regarded as the dependent variable; then, a discrete function f(τ) is constructed. We find the forward difference of f(τ), noted as $f^{\prime }\left(\tau \right)$, as shown in Figure 6.

Figure 6 reveals that, during the transitions from scale 2 to scale 3 or from scale 8 to scale 9, $f^{\prime }\left(\tau \right)$ exhibits the phenomenon of “zero crossing”—changing from positive to negative—across all evaluation metrics. For a discrete function, the occurrence of zero crossing corresponds to the local extremum point of its primitive function. Thus, τ = 3 and τ = 9 are identified as the local maximum points of f(τ), indicating that the maxima and extrema of model performance coincide. This suggests that the scale variation for the depression recognition model follows a certain pattern, rather than being random or disordered.

**Figure 7:**
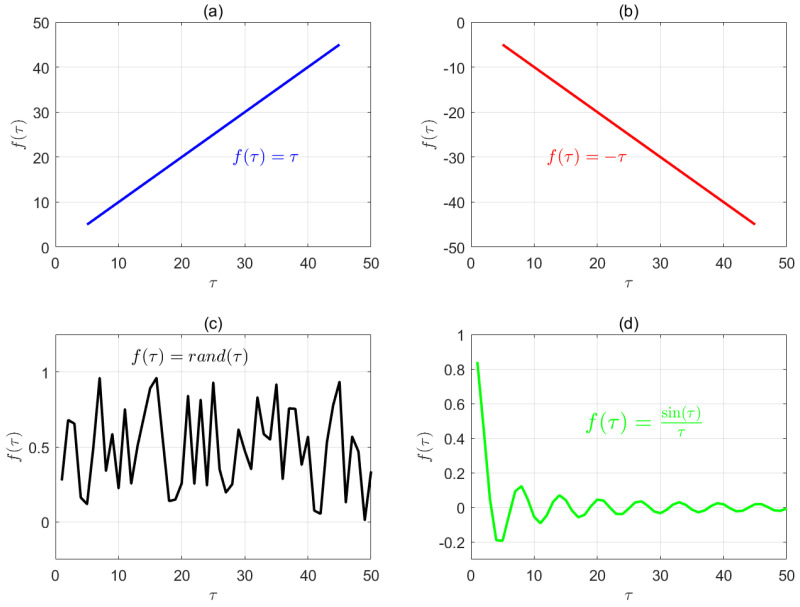
Four possible scenarios of the correlation between the temporal scale τ and model performance f(τ). (**a**) Positive correlation, corresponding to a monotonically increasing function. (**b**) Negative correlation, corresponding to a monotonically decreasing function. (**c**) No correlation, corresponding to a random function. (**d**) Specific correlation, i.e., nonlinear correlation. This could be understood as a relation where no monotonic function exists between two variables (factors), but the associated system exhibits a distinct pattern. Mathematically, this often manifests as some form of periodicity. In engineering applications or pattern recognition, this may indicate certain chaotic phenomena.

To further explain this, the concept of correlation is introduced. Figure 7 presents four types of functions, corresponding to the cases of a positive correlation, negative correlation, no correlation, and specific correlation, respectively. By analogy, if the model performance is positively correlated with the temporal scale, the multiscale analysis benefits depression recognition, and larger scales enhance this discriminative capability. Conversely, if the model performance is negatively correlated with the temporal scale, the multiscale analysis adversely affects depression recognition, and larger scales amplify the negative impact, reducing its discriminative power. If the model performance is uncorrelated with the temporal scale, the multiscale analysis becomes irrelevant for depression recognition, implying that the scale variation does not influence the model performance, or such effects are unquantifiable.

The fourth case, represented by the sinc function, corresponds to a “specific correlation” with the independent variable. It cannot be simply described as a positive or negative correlation but instead follows a distinct pattern. Analogously, the earlier discussion on the relation between the model performance and temporal scale implies the existence of such a pattern. Although it is challenging to depict this pattern mathematically in physiological systems (as most physiological signals are inherently stochastic processes [21]), this study attempts to demonstrate that, when considering multiscale analysis, there is a correlation between the model performance and temporal scale. Within a finite range of temporal scales, an optimal scale probably exists. Future research could build on this foundation to identify more statistical patterns and related applications.

### 4.2. Time Complexity

One crucial consideration when deploying depression recognition models in practice is the execution time, or, more specifically, the time consumed for feature extraction. The multiscale method would create an additional layer of complexity compared to the existing model. It is necessary to assess the computational cost.

The time complexity of the sample entropy algorithm is $o(N^{2})$ [11], and the time complexity of the permutation entropy algorithm is o(N) [12]. The basic idea of multiscale analysis is to calculate the entropy after the sequence is coarse-grained, so the factor affecting the time complexity is the sequence length *N*. We assume that the length of the original sequence is *L*, and the length of the reconstructed sequence is L/k at scale *k*. According to the algorithm flow described in Section 2.4, to calculate the RCMSE on scale τ, all scales of k∈[1,τ] should be considered, so the time complexity of RCMSE is

$$T_{1}\left(\tau \right)=o\left(L^{2}\right)+o\left({\left(\frac{L}{2}\right)}^{2}\right)+\cdots +o\left({\left(\frac{L}{\tau }\right)}^{2}\right).$$

To simplify the analysis, let $o\left(L^{2}\right)=aL^{2}$, where *a* is a constant coefficient. Then, the above formula becomes

(8) $$\begin{array}{cc} T_{1}\left(\tau \right) & =aL^{2}+a{\left(\frac{L}{2}\right)}^{2}+\cdots +a{\left(\frac{L}{\tau }\right)}^{2}\\ \; & =aL^{2}\sum_{k=1}^{\tau }\frac{1}{k^{2}}. \end{array}$$

Similarly, let o(L)=bL, where *b* is another constant. Thus, the time complexity of RCMPE is

(9) $$\begin{array}{cc} T_{2}\left(\tau \right) & =o\left(L\right)+o\left(\frac{L}{2}\right)+\cdots +o\left(\frac{L}{\tau }\right)\\ \; & =bL+\frac{bL}{2}+\cdots +\frac{bL}{\tau }\\ \; & =bL\sum_{k=1}^{\tau }\frac{1}{k}. \end{array}$$

Another subprocess influencing the overall computational complexity is coarse-graining, which can be decomposed into segmenting the sequence and then averaging each subsequence to obtain a reconstructed sequence. Referring to Figure 1 and (1), the length of each subsequence is *k*, and the total number of subsequences is L/k. Since the averaging process for each subsequence involves k−1 complex additions and one complex multiplication, the time complexity of coarse-graining is

(10) $$\begin{array}{cc} T_{3}\left(\tau \right) & =\sum_{k=1}^{\tau }\frac{L}{k}\left[c_{1}\left(k-1\right)+c_{2}\right]\\ \; & =L\sum_{k=1}^{\tau }\left[\frac{c_{1}\left(k-1\right)}{k}+\frac{c_{2}}{k}\right]\\ \; & =L\sum_{k=1}^{\tau }\left(c_{1}+\frac{c_{2}-c_{1}}{k}\right)\\ \; & =c_{1}L\tau +\left(c_{2}-c_{1}\right)L\sum_{k=1}^{\tau }\frac{1}{k}, \end{array}$$

where $c_{1}$ and $c_{2}$, respectively, denote the time required for a single complex addition and multiplication on a computer. Notably, if $c_{1}$ and $c_{2}$ are approximately equal, then $T_{3}\left(\tau \right)=c_{1}L\tau$, indicating a linear relationship between $T_{3}$ and τ.

The sum of (8)–(10) constitutes the overall time complexity, denoted as *E*, which can be expressed as

(11) $$\begin{array}{cc} E(\tau ) & =T_{1}\left(\tau \right)+T_{2}\left(\tau \right)+T_{3}\left(\tau \right)\\ \; & =aL^{2}\sum_{k=1}^{\tau }\frac{1}{k^{2}}+bL\sum_{k=1}^{\tau }\frac{1}{k}+c_{1}L\tau +\left(c_{2}-c_{1}\right)L\sum_{k=1}^{\tau }\frac{1}{k}\\ \; & =c_{1}L\tau +\left(b+c_{2}-c_{1}\right)L\sum_{k=1}^{\tau }\frac{1}{k}+aL^{2}\sum_{k=1}^{\tau }\frac{1}{k^{2}}. \end{array}$$

**Figure 8:**
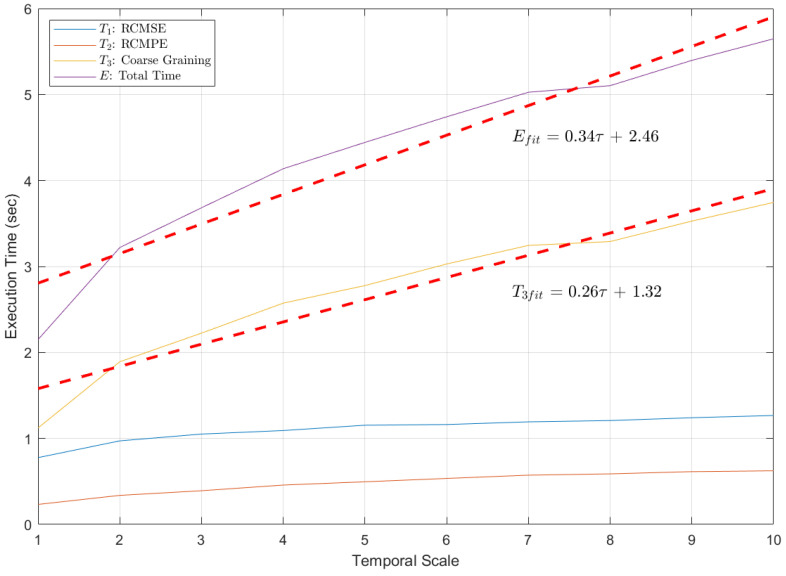
Execution times of different programming parts for 5 min single-channel EEG. Software: Windows 10 OS, MATLAB R2021b. Hardware: Intel(R) Core(TM) i5-8300H CPU @2.30 GHz.

Intuitively, as τ→∞, $c_{1}L\tau$ is a linear function, while $\sum \frac{1}{k}$ and $\sum \frac{1}{k^{2}}$ both behave as infinite series. Thus, the contribution of the latter two terms to E(τ) becomes negligible. However, it is impossible to set τ to infinity in reality. In fact, τ = 20 is almost the limit of engineering applications [8]. Therefore, the Profiler toolbox in MATLAB was utilized to conduct an algorithm complexity test. The execution times of the programs for feature extraction were recorded when τ∈[1,10], as shown in Figure 8.

As shown in Figure 8, the time complexity of all sub-algorithms increases with τ, which is straightforward to understand. Both $T_{1}$ and $T_{2}$ grow slowly with τ due to the properties of series expansion. In contrast, $T_{3}$ exhibits a linear relationship with τ, consistent with the previous derivation. Notably, $T_{3}$ accounts for a larger proportion of the execution time compared to $T_{1}$ and $T_{2}$, making it the dominant term in *E*. While this phenomenon is theoretically valid for large τ, it is also observed in practice for small τ. Consequently, *E* naturally exhibits a linear relationship with τ. This suggests that the complexity of the depression recognition model is linearly correlated with the temporal scale. One possible explanation is that, in (11), $c_{1}$ and $c_{2}$ are significantly larger than *a* and *b*. This also underscores the strong dependence of the literal performance of multiscale algorithms on the hardware capabilities.

Generally, the linear complexity is acceptable in practice, reinforcing the feasibility of applying multiscale analysis to depression recognition. Clinical diagnosis typically requires a prolonged waiting period. Moreover, depression screening based on EEG does not demand real-time performance, unlike epilepsy prediction or other cognitive tasks [22]. Given the discussion in Section 4.1, it is reasonable to conclude that, by accepting predictable computational costs, a stable improvement in model performance can be achieved.

### 4.3. Limitations and Prospects

The depression recognition models that have emerged in recent years were selected for a brief comparison, as shown in Table 8.

Similar to other EEG-based recognition tasks, the improvement in model performance primarily relies on the selection and combination of effective features, as well as the use of appropriate classifiers for model training. This study integrates the improved multiscale sample entropy and permutation entropy (RCMSE and RCMPE) as a feature vector, leveraging insights from previous research [17]. Although the model performance is satisfactory, there exists room for improvement. Firstly, in order to accurately assess the effect of the temporal scale on the algorithm, multidomain features were not constructed, which means that only two types of entropy metrics (nonlinear domain) were used as EEG features. Compared to more complex models [26], the proposed model may not be optimal. Multidomain features may lead to the problem of overfitting, although the dimensions of the feature vectors can be reduced by means of feature selection [27]. Therefore, future research on depression models should still consider the usage of multidomain features. Secondly, the application of deep learning techniques is highly valued. Although inconvenient to deploy on clinical devices, models based on deep learning generally exhibit better performance than conventional machine learning approaches [28].

Yun et al. [7] examined the temporal characteristics of depression-related EEG signals, but they did not explore the feasibility of model construction. This paper extends and validates some of the previous conclusions, namely that specific temporal scales prove to be more conducive to model development. It is noteworthy that the models constructed by different methods may not possess the same optimal scales. Changing the model configuration (applying different features or classifiers) does not guarantee that the optimal scale will coincide with that identified in existing work. However, based on the discussion in Section 4.1 and Section 4.2, it can be inferred that depression recognition models always identify the optimal scales with linear time complexity. Assuming that this conclusion holds for any algorithms involving multiscale analysis (such as the Lempel–Ziv Complexity [29]), subsequent research could extend beyond the optimal scales identified in this study and instead focus on re-determining the optimal scales following the specific algorithm. However, the reliability of this conclusion needs to be validated through more rigorous experimentation.

In addition, since the EEG signals of different brain regions were not analyzed statistically, but a mathematical analysis was performed instead, further neurological investigations are warranted. For instance, the correlation analysis of various brain regions on different temporal scales could be considered, in order to elucidate differences in brain functional connectivity [30]. The biomedical basis regarding how temporal scales affect information expression might be more enlightening than solely improving the model’s performance [31]. For instance, Jaworska et al. [6] investigated the relation between EEG variability and the efficacy of antidepressant treatments based on MSE. Notably, the impact of patients’ age was included in their work, enhancing the clinical application value. Relevant studies are also expected to provide new solutions for the control and treatment of depression. Lastly, since the data in our research only contained the EEG signals of major depressive disorder (MDD) patients and healthy controls, subsequent work could enrich the depression subtypes to make the recognition model more clinically meaningful [32].

Moreover, several considerations arise in the selection of the algorithm parameters. In this study, the parameters that were discussed included the window length and the range of temporal scales (i.e., the maximum temporal scale). These settings were determined with reference to the existing literature [7,18]. However, it is crucial to acknowledge a fundamental issue in multiscale analysis: the conflict between the data length requirements for coarse scales and the inconsistency of brain states over long signals. Coarser-scale analysis demands longer data segments (window length). However, excessively large windows could alter the brain states at finer scales, rendering the entropy estimation results unreliable. One potential solution is to perform “segmentation” after “coarse-graining”, ensuring that the signals across all scales share the same window length during entropy extraction. However, this approach introduces significant differences in the number of usable samples among scales, potentially leading to unpredictable effects on the statistical analysis or model training.

In addition, the electrodes can sometimes be regarded as parameters. For instance, Morabito et al. [33] proposed multivariate multiscale permutation entropy (MMSPE) to distinguish different stages of Alzheimer’s disease. This method computes the probabilities of permutation patterns across multiple electrodes simultaneously, yielding a form of joint entropy. MMSPE is also categorized as multiscale entropy. Unfortunately, due to data limitations, their study used a window length of 3 s and only extracted entropy values for scales 1 to 4, leaving group differences at larger window lengths or coarser scales unexplored. Nevertheless, the value of MMSPE should not be overlooked. If a high degree of correlation exists between the electrodes (Table 5), MMSPE can help to reduce redundant information. However, when the interelectrode correlation is uncertain (Table 6), it is suggested to apply this method with caution.

## 5. Conclusions

In this study, two multiscale entropy algorithms, RCMSE and RCMPE, were employed to extract EEG features, while four classifiers, LDA, LR, RBF-SVM, and KNN, were utilized for depression recognition experiments on the HUSM dataset. The model performance was evaluated across temporal scales ranging from 1 to 10. The experimental results revealed that scales 3 and 9 yielded the best performance among the various classifiers. The performance of the depression recognition model demonstrated a correlation with scale variation, suggesting that an optimal scale likely exists within a finite range. The execution time of the algorithm exhibited a linear relationship with the temporal scale. By accepting predictable increases in the computational time, a stable improvement in model performance can be achieved. Consequently, multiscale analysis is a feasible method for the building and optimization of a depression recognition model. Future research could benefit from statistical analyses across brain regions to provide further insights.

## Figures and Tables

**Table 1:** Classification accuracy for different temporal scales and classifiers.

Classifier	τ = 1	τ = 2	τ = 3	τ = 4	τ = 5	τ = 6	τ = 7	τ = 8	τ = 9	τ = 10
LDA	85.27%	83.84%	86.39%	85.59%	84.95%	84.00%	85.11%	85.51%	85.59%	85.83%
LR	83.84%	83.84%	87.26%	85.51%	83.92%	83.52%	84.32%	84.55%	84.39%	84.71%
RBF-SVM	94.11%	95.46%	95.46%	95.22%	94.59%	94.43%	95.14%	95.30%	95.46%	95.14%
KNN	93.63%	94.27%	94.59%	94.27%	93.07%	93.31%	94.35%	95.06%	**96.42%**	94.51%

**Table 2:** Sensitivity for different temporal scales and classifiers.

Classifier	τ = 1	τ = 2	τ = 3	τ = 4	τ = 5	τ = 6	τ = 7	τ = 8	τ = 9	τ = 10
LDA	78.01%	75.53%	80.31%	79.35%	78.20%	77.25%	79.54%	79.16%	79.73%	80.31%
LR	77.06%	78.20%	82.79%	80.31%	78.97%	78.97%	79.16%	79.35%	79.73%	80.50%
RBF-SVM	89.87%	91.01%	93.50%	93.31%	91.20%	**94.07%**	93.69%	**94.07%**	93.88%	93.69%
KNN	90.06%	89.87%	90.06%	90.25%	87.57%	89.67%	91.20%	92.54%	93.88%	91.20%

**Table 3:** Specificity for different temporal scales and classifiers.

Classifier	τ = 1	τ = 2	τ = 3	τ = 4	τ = 5	τ = 6	τ = 7	τ = 8	τ = 9	τ = 10
LDA	90.45%	89.77%	90.72%	90.04%	89.77%	88.81%	89.09%	90.04%	89.77%	89.77%
LR	88.68%	87.86%	90.45%	89.22%	87.45%	86.77%	87.99%	88.27%	87.72%	87.72%
RBF-SVM	97.14%	**98.64%**	96.86%	96.59%	97.00%	94.68%	96.18%	96.18%	96.59%	96.18%
KNN	96.18%	97.41%	97.82%	97.14%	97.00%	95.91%	96.59%	96.86%	98.23%	96.86%

**Table 4:** F1-score for different temporal scales and classifiers.

Classifier	τ = 1	τ = 2	τ = 3	τ = 4	τ = 5	τ = 6	τ = 7	τ = 8	τ = 9	τ = 10
LDA	0.815	0.796	0.831	0.821	0.812	0.801	0.816	0.820	0.822	0.825
LR	0.799	0.801	0.844	0.822	0.804	0.800	0.808	0.811	0.810	0.814
RBF-SVM	0.927	0.944	0.945	0.942	0.933	0.934	0.941	0.943	0.945	0.941
KNN	0.922	0.929	0.933	0.929	0.913	0.918	0.931	0.940	**0.956**	0.933

**Table 5:** The group difference (*p*-values) of RCMSE.

Electrode	τ = 1	τ = 2	τ = 3	τ = 4	τ = 5	τ = 6	τ = 7	τ = 8	τ = 9	τ = 10
Fp1										
F3	$1.4\times 10^{-2}$	$4.7\times 10^{-3}$	$2.5\times 10^{-3}$	$1.6\times 10^{-3}$	$1.2\times 10^{-3}$	$1.0\times 10^{-3}$	$8.3\times 10^{-4}$	$7.2\times 10^{-4}$	$6.3\times 10^{-4}$	$5.7\times 10^{-4}$
C3										
P3	$9.4\times 10^{-5}$	$3.4\times 10^{-4}$	$7.4\times 10^{-4}$	$1.2\times 10^{-3}$	$1.6\times 10^{-3}$	$2.0\times 10^{-3}$	$2.5\times 10^{-3}$	$3.0\times 10^{-3}$	$3.5\times 10^{-3}$	$3.9\times 10^{-3}$
O1	$6.8\times 10^{-13}$	$2.5\times 10^{-12}$	$7.3\times 10^{-12}$	$1.5\times 10^{-11}$	$2.6\times 10^{-11}$	$3.8\times 10^{-11}$	$5.1\times 10^{-11}$	$6.6\times 10^{-11}$	$8.4\times 10^{-11}$	$1.0\times 10^{-10}$
F7	$3.0\times 10^{-3}$	$8.1\times 10^{-4}$	$4.6\times 10^{-4}$	$3.5\times 10^{-4}$	$3.0\times 10^{-4}$	$2.6\times 10^{-4}$	$2.4\times 10^{-4}$	$2.2\times 10^{-4}$	$2.0\times 10^{-4}$	$1.9\times 10^{-4}$
T3	$7.1\times 10^{-12}$	$5.4\times 10^{-11}$	$1.5\times 10^{-10}$	$2.6\times 10^{-10}$	$4.1\times 10^{-10}$	$5.7\times 10^{-10}$	$7.4\times 10^{-10}$	$9.1\times 10^{-10}$	$1.1\times 10^{-9}$	$1.2\times 10^{-9}$
T5	$6.3\times 10^{-11}$	$2.5\times 10^{-10}$	$6.4\times 10^{-10}$	$1.2\times 10^{-9}$	$1.8\times 10^{-9}$	$2.4\times 10^{-9}$	$3.2\times 10^{-9}$	$4.0\times 10^{-9}$	$4.8\times 10^{-9}$	$5.7\times 10^{-9}$
Fz	$6.1\times 10^{-5}$	$1.1\times 10^{-5}$	$3.2\times 10^{-6}$	$1.5\times 10^{-6}$	$8.8\times 10^{-7}$	$5.7\times 10^{-7}$	$4.0\times 10^{-7}$	$3.0\times 10^{-7}$	$2.4\times 10^{-7}$	$1.9\times 10^{-7}$
Fp2	$1.5\times 10^{-3}$	$4.6\times 10^{-4}$	$2.3\times 10^{-4}$	$1.7\times 10^{-4}$	$1.3\times 10^{-4}$	$1.1\times 10^{-4}$	$1.0\times 10^{-4}$	$9.2\times 10^{-5}$	$8.4\times 10^{-5}$	$7.7\times 10^{-5}$
F4										
C4										
P4	$7.1\times 10^{-5}$	$3.1\times 10^{-4}$	$7.1\times 10^{-4}$	$1.2\times 10^{-3}$	$1.8\times 10^{-3}$	$2.3\times 10^{-3}$	$2.9\times 10^{-3}$	$3.4\times 10^{-3}$	$4.0\times 10^{-3}$	$4.6\times 10^{-3}$
O2	$4.9\times 10^{-11}$	$1.1\times 10^{-10}$	$2.3\times 10^{-10}$	$3.8\times 10^{-10}$	$5.4\times 10^{-10}$	$6.8\times 10^{-10}$	$8.3\times 10^{-10}$	$9.8\times 10^{-10}$	$1.1\times 10^{-9}$	$1.3\times 10^{-9}$
F8										
T4	$5.0\times 10^{-3}$	$1.6\times 10^{-2}$	$2.7\times 10^{-2}$	$3.6\times 10^{-2}$	$4.3\times 10^{-2}$	$5.0\times 10^{-2}$				
T6	$1.7\times 10^{-6}$	$9.5\times 10^{-6}$	$2.2\times 10^{-5}$	$3.8\times 10^{-5}$	$5.4\times 10^{-5}$	$7.0\times 10^{-5}$	$8.7\times 10^{-5}$	$1.0\times 10^{-4}$	$1.2\times 10^{-4}$	$1.3\times 10^{-4}$
Cz										
Pz	$1.3\times 10^{-4}$	$5.4\times 10^{-4}$	$1.2\times 10^{-3}$	$2.0\times 10^{-3}$	$2.7\times 10^{-3}$	$3.4\times 10^{-3}$	$4.2\times 10^{-3}$	$5.1\times 10^{-3}$	$5.9\times 10^{-3}$	$6.6\times 10^{-3}$

**Table 6:** The group difference (*p*-values) of RCMPE.

Electrode	τ = 1	τ = 2	τ = 3	τ = 4	τ = 5	τ = 6	τ = 7	τ = 8	τ = 9	τ = 10
Fp1	$3.2\times 10^{-3}$	$5.5\times 10^{-8}$	$2.2\times 10^{-2}$				$1.8\times 10^{-3}$	$2.6\times 10^{-5}$	$1.4\times 10^{-5}$	$5.8\times 10^{-5}$
F3	$3.3\times 10^{-4}$		$2.6\times 10^{-4}$	$4.1\times 10^{-4}$	$1.9\times 10^{-6}$	$5.1\times 10^{-11}$	$3.5\times 10^{-15}$	$1.9\times 10^{-17}$	$3.2\times 10^{-18}$	$2.0\times 10^{-16}$
C3	$3.9\times 10^{-9}$		$1.0\times 10^{-6}$	$6.0\times 10^{-5}$	$7.4\times 10^{-5}$	$9.2\times 10^{-8}$	$6.0\times 10^{-11}$	$4.2\times 10^{-11}$	$1.0\times 10^{-11}$	$6.3\times 10^{-11}$
P3	$2.9\times 10^{-4}$		$7.8\times 10^{-3}$				$3.8\times 10^{-3}$	$8.0\times 10^{-3}$	$3.1\times 10^{-3}$	$6.6\times 10^{-3}$
O1	$4.4\times 10^{-3}$	$3.5\times 10^{-6}$		$1.0\times 10^{-4}$	$2.6\times 10^{-6}$	$7.0\times 10^{-5}$	$1.7\times 10^{-2}$	$7.6\times 10^{-3}$	$3.6\times 10^{-2}$	$3.0\times 10^{-2}$
F7	$2.8\times 10^{-3}$	$6.3\times 10^{-3}$		$3.8\times 10^{-2}$	$3.9\times 10^{-4}$	$6.4\times 10^{-8}$	$9.8\times 10^{-12}$	$4.3\times 10^{-14}$	$5.8\times 10^{-15}$	$3.9\times 10^{-14}$
T3		$6.3\times 10^{-8}$	$1.5\times 10^{-2}$	$2.1\times 10^{-3}$	$1.6\times 10^{-3}$	$2.9\times 10^{-2}$				
T5	$5.6\times 10^{-3}$	$1.4\times 10^{-8}$		$4.0\times 10^{-4}$	$5.1\times 10^{-4}$	$1.3\times 10^{-2}$				
Fz	$6.9\times 10^{-18}$	$2.3\times 10^{-2}$	$1.2\times 10^{-12}$	$2.3\times 10^{-12}$	$4.7\times 10^{-11}$	$2.3\times 10^{-18}$	$1.6\times 10^{-25}$	$5.0\times 10^{-26}$	$9.2\times 10^{-28}$	$1.7\times 10^{-26}$
Fp2	$1.6\times 10^{-13}$	$5.7\times 10^{-7}$				$9.4\times 10^{-4}$	$1.5\times 10^{-7}$	$1.2\times 10^{-10}$	$8.4\times 10^{-12}$	$6.1\times 10^{-11}$
F4	$8.2\times 10^{-10}$		$1.9\times 10^{-3}$	$7.8\times 10^{-3}$	$3.8\times 10^{-3}$	$1.5\times 10^{-6}$	$1.8\times 10^{-10}$	$2.0\times 10^{-11}$	$1.2\times 10^{-12}$	$9.0\times 10^{-12}$
C4	$8.6\times 10^{-8}$		$2.4\times 10^{-3}$	$1.1\times 10^{-2}$	$3.8\times 10^{-3}$	$2.2\times 10^{-5}$	$9.1\times 10^{-8}$	$2.6\times 10^{-8}$	$8.4\times 10^{-9}$	$4.8\times 10^{-8}$
P4	$2.7\times 10^{-4}$		$1.1\times 10^{-3}$			$3.1\times 10^{-2}$	$4.0\times 10^{-4}$	$1.3\times 10^{-3}$	$2.2\times 10^{-4}$	$3.6\times 10^{-4}$
O2	$1.2\times 10^{-2}$	$1.4\times 10^{-4}$	$3.6\times 10^{-2}$	$5.5\times 10^{-5}$	$1.7\times 10^{-5}$	$3.2\times 10^{-5}$	$2.6\times 10^{-3}$	$1.6\times 10^{-3}$	$4.6\times 10^{-3}$	$3.2\times 10^{-3}$
F8	$1.4\times 10^{-12}$	$2.9\times 10^{-8}$				$6.1\times 10^{-4}$	$2.6\times 10^{-8}$	$1.6\times 10^{-10}$	$2.7\times 10^{-12}$	$6.4\times 10^{-12}$
T4	$9.5\times 10^{-4}$	$3.3\times 10^{-3}$					$4.1\times 10^{-3}$	$5.7\times 10^{-4}$	$1.2\times 10^{-4}$	$3.1\times 10^{-4}$
T6	$3.8\times 10^{-3}$		$2.1\times 10^{-2}$				$1.9\times 10^{-2}$	$1.8\times 10^{-2}$	$2.9\times 10^{-3}$	$3.1\times 10^{-3}$
Cz	$2.5\times 10^{-11}$		$3.6\times 10^{-6}$	$8.5\times 10^{-5}$	$2.1\times 10^{-4}$	$1.1\times 10^{-7}$	$8.6\times 10^{-11}$	$6.3\times 10^{-11}$	$1.2\times 10^{-11}$	$1.0\times 10^{-10}$
Pz	$1.7\times 10^{-11}$		$2.2\times 10^{-3}$			$2.1\times 10^{-2}$	$3.3\times 10^{-4}$	$8.6\times 10^{-4}$	$2.4\times 10^{-4}$	$5.7\times 10^{-4}$

Hypothesis testing method: independent-samples *t*-test, *α* = 0.05. *p*-values greater than 0.05 are not included in the table.

**Table 7:** The optimal temporal scales of the proposed model.

Accuracy	Sensitivity	Specificity	F1-Score
τ = 9 in KNN	τ = 6/8 in SVM	τ = 2 in SVM	τ = 9 in KNN
τ = 3 (average)	τ = 9 (average)	τ = 3 (average)	τ = 3 (average)

**Table 8:** Comparison with existing models.

Authors	Year	Feature	Classifier	Accuracy
Yun et al. [7]	2021	RCMPE	LR	75% (LOOCV ^1^)
Čukić et al. [14]	2020	HFD, Sample Entropy	MLP, LR, SVM, DT, RF, Naive Bayes	90.24–97.56%
Jiang et al. [23]	2021	TCSP + Differential Entropy	LR, SVM, KNN	84–85.7%
Liu et al. [24]	2022	Band Power, LZC, DFA	LDA, LR, SVM	89.29% (average)
Avots et al. [19]	2022	Relative Band Power, Alpha Power Variability, Spectral Asymmetry Index, HFD, LZC, DFA	SVM, LDA, DT, Naive Bayes	80–95%
Yang et al. [25]	2023	LZC	SVM, KNN, DT	94.03% (highest)
The proposed method	-	RCMSE, RCMPE	LDA, LR, RBF-SVM, KNN	96.42% (highest), 90.92% (average)

^1^ Leave-one-out cross-validation, which is a specific case of k-fold cross-validation (Section 2.8) where k = n.

## Data Availability

The original data presented in the study are openly available on FigShare at https://figshare.com/articles/dataset/EEG_Data_New/4244171 (accessed on 17 December 2024).
